# Randomly amplified polymorphic DNA analysis of *Staphylococcus chromogenes* isolated from bovine and bubaline mastitis in Karnataka

**DOI:** 10.14202/vetworld.2021.285-291

**Published:** 2021-01-30

**Authors:** P. Sheela, Malathi Shekar, Shrikrishna Isloor, D. Rathnamma, B. M. Veeregowda, M. L. Satyanarayana, S. Sundareshan, B. E. Shambulingappa, Nagendra R. Hegde

**Affiliations:** 1 Department of Veterinary Microbiology, Veterinary College, Shivamogga, Karnataka Veterinary Animal and Fisheries Sciences University, Shivamogga, Karnataka, India; 2 Bioinformatics Centre, Department of Aquatic Animal Health Management, College of Fisheries, Mangaluru, Karnataka Veterinary Animal and Fisheries Sciences University, Mangaluru, Karnataka, India; 3 Department of Microbiology, Veterinary College, Bengaluru, Karnataka Veterinary Animal and Fisheries Sciences University, Bengaluru, Karnataka, India; 4 Department of Pathology, Veterinary College, Bengaluru, Karnataka Veterinary Animal and Fisheries Sciences University, Bengaluru, Karnataka, India; 5 National Institute of Animal Biotechnology, Gachibowli, Hyderabad, Telangana, India

**Keywords:** mastitis, methicillin resistance, non-aureus staphylococci, randomly amplified polymorphic DNA, *Staphylococcus chromogenes*

## Abstract

**Background and Aim:**

In recent times, non-aureus staphylococci (NAS) have emerged as the major organisms isolated from mastitis cases in dairy animals, with a predominance of *Staphylococcus epidermidis* and *Staphylococcus chromogenes*. As compared to *Staphylococcus aureus*, much less is known about the molecular types or the spatiotemporal epidemiology of these NAS species. In the present study, randomly amplified polymorphic DNA (RAPD) was employed to detect genetic polymorphisms, intraspecies diversity, and epidemiology of *S. chromogenes* strains (n=37) isolated from bovine and bubaline mastitis cases in the state of Karnataka.

**Materials and Methods:**

Thirty-seven *S. chromogenes* isolates (14 from bovines and 23 from bubaline) isolated from subclinical mastitis cases, from organized and unorganized sectors, were subjected to RAPD typing. Further, methicillin resistance was determined by cefoxitin disk diffusion method.

**Results:**

The amplified DNA fragments ranged from 150 to 3000 base pairs and yielded several RAPD profiles. Further analysis using Digital Image Correlation Engine correlation coefficient and UPGMA method showed that the 37 isolates could be classified into 12 distinct RAPD types (A to L) at 62% similarity (*D*=0.889). Four of the most predominant RAPD types, B, A, C, and E, in that order, and together, represented 65% of the isolates. High diversity was observed among the isolates both within farms and between geographic locations. Most of the isolates exhibited methicillin resistance. This is the first such report from India.

**Conclusion:**

In the absence of defined multilocus sequence type protocols or sufficient sequences available in the public domain, RAPD can be employed to determine genetic diversity of *S. chromogenes* isolates.

## Introduction

Bovine mastitis is an important disease which affects productivity in dairy animals, thereby directly or indirectly affecting farmers’ income and the economy of the country [1]. In India, *Staphylococcus* species constitute the main etiological agents of mastitis in cattle and buffaloes [2]; however, of late, non-aureus staphylococci (NAS) have been increasingly isolated [3]. The most predominantly reported NAS causing persistent intramammary infections and high somatic cell counts (SCCs) is *Staphylococcus chromogenes* [4-8].

Molecular epidemiological studies can contribute to the understanding of source, transmission routes, disease causation and outcome, and prognosis as well as to the understanding of mechanisms of host adaptation of pathogens. For *Staphylococcus aureus*, several typing methods have been used, and these include ribotyping, random amplification of polymorphic DNA (RAPD), pulsed-field gel electrophoresis (PFGE), multilocus sequence typing, *spa* typing, and multiple locus variable number tandem repeat analysis [9]. For NAS species, strain typing methods such as PFGE, amplified fragment length polymorphism, RAPD [10,11], and whole-genome sequencing [12] have been employed. The polymerase chain reaction (PCR)-based DNA fingerprinting method, RAPD, which is a rapid, discriminative, easily performable, and interpretable analytical assay, has been successfully used to detect polymorphisms of both *S. aureus* and NAS strains isolated from bovine mastitis [11,13-17].

There is very little information on the genotypic profiles and antimicrobial resistance of mastitis-associated *S. chromogenes* isolates from bovine mastitis cases in India. Hence, the present work was undertaken to investigate the intraspecies diversity and their genetic relationship using RAPD as well as methicillin resistance of mastitis-associated *S. chromogenes* isolates.

## Materials and Methods

### Ethical approval

Ethical committee approval was not required as the study did not involve any animal experimental work.

### Study area and period

The bacterial isolates comprised those isolated previously from subclinical mastitis (SCM) cases in organized and unorganized sectors, from samples collected from different geographical locations [in and around Bengaluru, (Tumakuru, Chennapatna, Ramanagara, near Hosur), Dharwad, and Bidar] between June 2009 and January 2013 (Table-1) from bovines and bubaline. Species confirmation by PCR was done and then RAPD analysis on *S. chromogenes* isolates were carried out in the month of september 2019.

**Table-1:** Diversity of RAPD types and methicillin resistance in different locations.

S. No.	Locations	Sector	Source	No. of isolates	RAPD types and methicillin resistance*
1	Bengaluru	Organized	Bovine	4	E (Bo32-R) F (Bo61-S, Bo62-S) J (Bo46 -S)
2	Bidar	Organized and unorganized	Bovine	6	E (Bo139-S), Bo146-R) J (Bo105-S, Bo107-R, Bo117-R) L(Bo157-R)
3	Channapatna	Unorganized	Bubaline	12	A (Bu117-R) B (Bu74-S, Bu75-S, Bu 90-R, Bu 91-R, Bu 99-R) C (Bu101-R) D (Bu79-S,102-R) E (Bu95-R) G (Bu92-S) H (Bu93-S)
4	Dharwad	Organized	Bubaline	8	A (Bu35-R, Bu 45-R) B (Bu30-R, Bu 42-R, Bu 43-R, Bu 46-R) C (Bu29-R, Bu 41-R)
5	Near Hosur	Unorganized	Bubaline	1	I (Bu59-S)
6	Ramanagara	Unorganized	Bubaline	2	C (Bu 105-S, Bu 119-R)
7	Tumakuru	Unorganized	Bovine	4	A (Bo26-S, Bo30-R) E (Bo23-S) K(Bo20-R)

* Isolate IDs followed by its resistance (R) or sensitivity (S) to methicillin are given in the parenthesis. RAPD=Randomly amplified polymorphic DNA

### Bacterial isolates used in the study

Following aseptic precautions, a pooled quarter milk sample was collected after discarding the initial few squirts of milk from each animal. Each animal was only sampled once. The milk was subjected to SCC, electrical conductivity (EC), California mastitis test, and bromothymol blue strip test. For declaring SCM in cows, the cutoff values used for SCC and EC were 5×10^5^ cells/mL and 6.5 mS/cm, respectively [18]. In buffaloes, the values were 2×10^5^ cells/mL and 3.8 mS/cm, respectively [3]. NAS were isolated following standard microbiological methods and had been maintained in the Department of Veterinary Microbiology, Karnataka Veterinary Animal and Fisheries Sciences University, Bengaluru.

### Extraction of genomic DNA

The genomic DNA of *Staphylococcus* isolates was extracted from an overnight culture, using a bacterial DNA extraction kit (Uniflex™ DNA Isolation Kit_,_ Genei™, Bengaluru, India), following the manufacturer’s instructions. The integrity of genomic DNA was assessed by 2% agarose gel electrophoresis and visualizing the bands through UV transillumination. The DNA samples were frozen at −20°C.

### Identification of the species of the isolates

The isolates were confirmed as *S. chromogenes* based on colony morphology (size and color), hemolytic pattern, Gram’s staining, and catalase and coagulase tests. Further confirmation was accomplished by PCR. For *S. chromogenes*, 303 base pairs (bp) region of the *sod*A gene [18] was amplified using the primer pairs 5’-CGTGACTAAGTTAAACGATGCAG-3’ (forward) and 5’-CCATTATTTACAACGAGCCATG-3’ (reverse). The reaction mixture (25 μL) contained 2.5 μL of 10× *Taq* buffer, 1 U of *Taq* DNA polymerase, 25 mM of each of the dNTPs, 10 pmoles/μL of each primer, 3 μL of template DNA, and 16.3 μL of nuclease-free water. The reaction was carried out with an initial denaturation step at 94°C for 5 min, followed by 30 cycles at 94°C for 30 s, 60°C for 30 s, and 72°C for 30 s, with a final extension step at 72°C for 10 min. The products were resolved by electrophoresis on 2% agarose gel, stained with ethidium bromide, and imaged using a gel documentation system (Gelstan 4×, The Medicare Scientific Supplies, India). The PCR products were sequenced (Eurofins) and subjected to bioinformatics analysis for species confirmation. In this study, 37 isolates (14 from bovines and 23 from bubaline) identified and confirmed as *S. chromogenes* were selected and subjected further to antimicrobial susceptible testing and RAPD analysis.

### Antimicrobial susceptibility testing to determine methicillin resistance

All the isolates were tested for methicillin resistance using cefoxitin disks (30 mg), by employing the Kirby–Bauer disk diffusion method [19], and the isolates were classified either as resistant or sensitive according to the guidelines recommended by the Clinical Laboratory Standards Institute [20].

### RAPD analysis

The RAPD typing was performed as described by Piessens *et al*. [11] with some modifications. The 23 bp primer 5′-AGTGAATTCGCGGT GAGATGCCA-3′ (D11344) was used, and RAPD-PCR was carried out in a 50 μL reaction mixture consisting of 25 μL of PCR master mix (Genei™_,_ Bengaluru), 1 μL of primer (10 μM), 1 μL of template DNA, and 23 μL of nuclease-free water. The cycling program consisted of four cycles of 94°C, 5 min; 36°C, 5 min; and 72°C, 5 min; followed by 30 cycles of 94°C, 1 min; 36°C, 1 min; and 72°C, 2 min, and then 72°C, 10 min. The fragments were separated by electrophoresing at 60 volts for 4 h on 2% agarose gel prepared in 1× Tris-Borate-EDTA buffer. The gels were stained with ethidium bromide and visualized using the gel documentation system.

The RAPD patterns were analyzed using Gel compare II version 2.5 (Applied Maths, St-Martens-Latem, Belgium). The similarity of profiles was calculated based on Digital Image Correlation Engine correlation coefficient and clustered using the unweighted pair group method with arithmetic mean (UPGMA) method. The discriminatory index (*D*) was calculated according to Hunter and Gaston [21]. The relationship between clustered groups was expressed as percentage similarity and presented as a dendrogram.

## Results

### Confirmation of S. chromogenes isolates by PCR

All the 37 isolates subjected to species-specific PCR yielded the expected 303 bp amplicon, and nucleotide sequencing confirmed the isolates to be *S. chromogenes*.

### Determination of methicillin resistance of S. chromogenes isolates

Fourteen of the 37 *S. chromogenes* isolates were found to be sensitive to methicillin while the remaining were resistant. All the isolates from Dharwad were resistant. Isolates from Bengaluru and Hosur were predominantly sensitive, whereas isolates from Bidar and Channapatna were predominantly resistant. In Ramanagara and Tumakuru, isolates were equally distributed between sensitive and resistant types. The proportion of resistant isolates was 50% (n=7) in bovines and 70% (n=16) in bubaline.

### RAPD and clustering analyses for S. chromogenes

**Figure-1:**
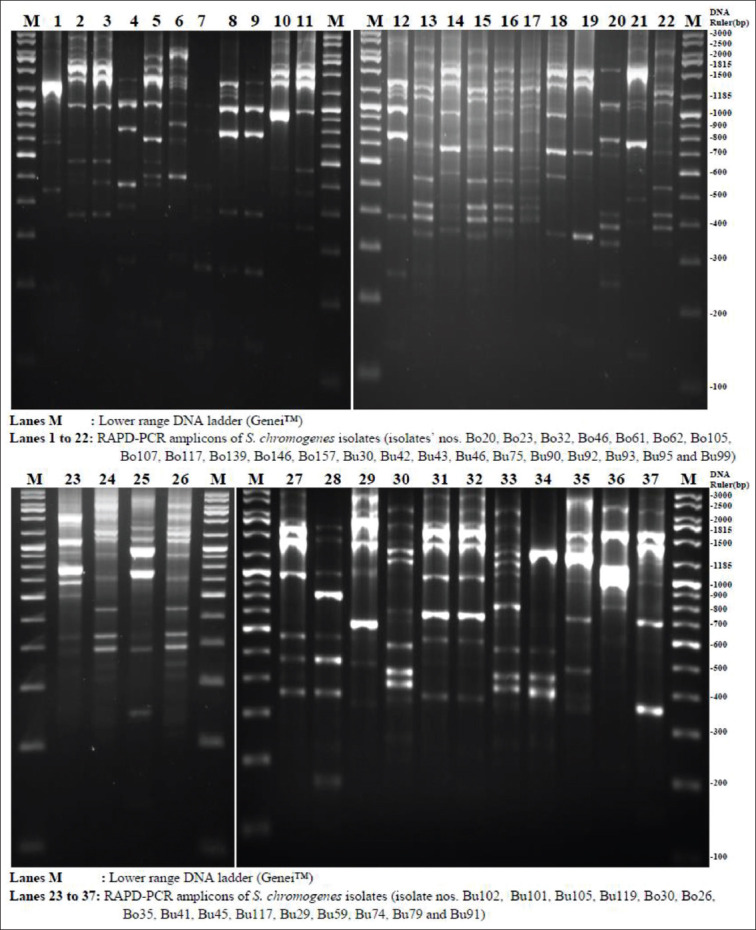
Randomly amplified polymorphic DNA polymerase chain reaction of *Staphylococcus chromogenes* isolates.

**Figure-2:**
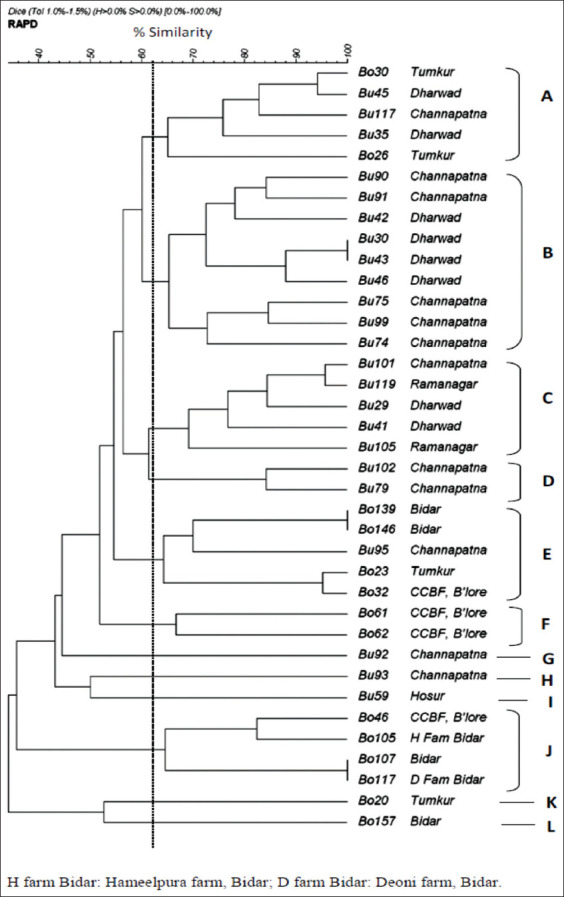
Dendrogram derived from the randomly amplified polymorphic DNA of *Staphylococcus chromogenes* isolates.

From the 37 isolates, the RAPD-PCR generated 7-19 fragments with DNA size ranging from 150 to 3000 bp (Figure-1). Analysis of RAPD profiles at 62% similarity revealed 12 types (A through L). A dendrogram generated based on the similarity matrix is presented (Figure-2 and Table-2). The most predominant profile type was B followed by A, C, and E, and they together represented 65% (24/37) of the isolates. Type B was characterized by the presence of 8-19 amplimers ranging from 320 to 2800 bp. Type A contained 9 or 11 amplimers ranging from 160 to 3000 bp. Type C consisted of 11-17 amplimers ranging from 210 to 2700 bp (Table-2 and Figure-2).

**Table-2:** Randomly amplified polymorphic DNA cluster analysis of the mastitic *Staphylococcus chromogenes* isolates.

S. No.	Cluster ID	Band size (base pairs)	No. of bands	Source of the isolates
1	A	160-3000	9-11	Bovine and bubaline
2	B	320-2800	8-19	Bubaline
3	C	210-2700	11-17	Bubaline
4	D	260-2400	9-15	Bubaline
5	E	350-2800	9-11	Bovine and bubaline
6	F	150-2100	9-13	Bovine
7	G	370-1600	7	Bubaline
8	H	260-1600	9	Bubaline
9	I	360-2400	10	Bubaline
10	J	130-1300	7-9	Bovine
11	K	190-3000	9	Bovine
12	L	240-2400	9	Bovine

Bovine isolates clustered in the RAPD types F, J, K, and L while bubaline isolates clustered under B, C, D, G, H, and I types. Types A and E consisted of five isolates each from bovine and bubaline herds (Table-2 and Figure-2).

Region-wise clustering of RAPD types revealed that types A and C contained isolates from three geographical locations, whereas type E contained five isolates from four locations. Type A contained five isolates from bovine herds of Tumakuru and bubaline herds of Channapatna and Dharwad. Type B consisted of nine isolates, all from bubaline herds of Channapatna and Dharwad. About 50% of the isolates (n=4) from Dharwad clustered under type B (Table-1 and Figure-2). Type C consisted of five isolates restricted to bubaline herds (Channapatna, Dharwad, and Ramanagara) (Table-1 and Figure-2). Types D and F contained two isolates each from Channapatna and Bengaluru, respectively. The types G and H contained one isolate each and both were from Channapatna. The other two types, K and L, contained one isolate each from Bidar and Tumakuru, respectively. Type J contained three isolates from Bidar and one from Bengaluru. Type I contained a solitary isolate from Hosur.

Highly diverse types were found in Channapatna, Tumakuru, and Bengaluru. Bubaline isolates (n=12) from Channapatna clustered under seven different RAPD types, and bovine isolates from Tumakuru and Bengaluru (n=4 each) clustered into three types. In Bengaluru, all the four isolates from a single farm clustered in three different RAPD types, whereas less diverse types were found in Bidar, Dharwad, and Ramanagara. In Bidar, all three bovine isolates from organized farms clustered under a single type (J) and two of the three isolates from unorganized farms clustered under a single type (E) (Table-2 and Figure-2).

## Discussion

Among the NAS, *S. chromogenes* is the most predominant pathogen associated with mastitis in India and other countries [4-8]. In the present study, genetic diversity of bovine and bubaline mastitis-associated *S. chromogenes* was evaluated by RAPD typing, a method which has been shown earlier to be useful in the discrimination of *S. chromogenes* isolates [17].

In the current study, 62% of *S. chromogenes* isolates were found to be resistant to methicillin, with a preponderance (70%) in bubaline isolates, whereas resistant and sensitive isolates were almost equally distributed in bovines. This proportion is much higher than 0% or 8.2% reported for bovine mastitis in Dutch dairy cows [5,22,23]. On the other hand, while no prevalence data have been presented, two *mec*A^+^ isolates, each of *S. chromogenes* and *Staphylococcus Epidermidis*, were reported from samples (n=150) of raw bovine milk, and ovine and caprine cheese in Northwest Spain [24].

The RAPD typing has been employed before for characterizing bovine mastitis-associated *S. aureus* isolates. In a study conducted in the USA and the Republic of Ireland [25], majority of *S. aureus* isolates from cows in Ireland had RAPD profiles similar to those identified for the isolates recovered from cows in the USA. While these data suggest that bovine mastitis is caused by a few clones of *S*. *aureus* which have a broad geographic dissemination, different RAPD types were identified in *S. aureus* isolates both within the herds and among different farms in China [16]. In any case, RAPD has not been popularly applied for molecular fingerprinting of livestock *S. aureus* isolates because of the wider usage of other typing methods.

The 37 *S. chromogenes* obtained from seven geographic regions in Karnataka clustered into 12 groups (A-J). Among these, 65% of the isolates clustered in four predominant RAPD types, namely, B, A, C, and E. Similar observations were made with large Chinese dairy farms in which eight distinct RAPD types (A-H) were observed, with 73% of the isolates being represented by the most prevalent types A and F [17]. This also corroborates the highly conserved patterns observed by PFGE in *S. chromogenes* isolates obtained from cows and pigs [26]. Analysis of genetic heterogeneity and diversity can be used to track the movement of pathogens as well as to associate with pathogenesis. A study with *S. chromogenes* isolates (n=28) causing bovine intramammary infections showed limited genotypic heterogeneity by RAPD both within and between Flemish dairy herds, reflecting high genetic conservation within the species [11]. Importantly, it was found that isolates from the environment displayed largely the same genotypes as those from milk, suggesting that they were more udder adapted. On the other hand, the isolates studied by us were more diverse both within the farms and between the locations, except for the bubaline isolates from Ramanagara where both of them belonged to type C, and in Dharwad, where 50% of the isolates belonged to type B (Table-1). Diversity was also observed with 66 *S. chromogenes* isolates obtained from 30 animals from three dairy herds of the USA, where 33 different PFGE patterns were observed indicating genetic diversity in strains even when multiple sampling was done [10]. However, it is difficult to generalize the results or compare the RAPD or PFGE types between distant geographical locations due to the small number of studies relating *S. chromogenes*.

Most of the isolates from the predominant RAPD types (A, B, C, and E) in the present study were methicillin-resistant except for isolates Bo26 in type A, Bu74 and Bu75 in type B, Bu105 in type C, and Bo23 and Bo139 in type E, all of which were sensitive.

## Conclusion

This is the first study from India with respect to molecular fingerprinting of mastitis-associated *S. chromogenes* isolates. In the absence of protocols for other typing methods or the availability of sufficient genome sequences, RAPD-PCR can be employed as a tool to determine the diversity of *S. chromogenes* isolates. However, a large number of isolates both from the environment and milk need to be analyzed to track transmission and dissemination events.

## Authors’ Contributions

PS, SI, and NRH designed the study. PS performed molecular typing of isolates by RAPD under the supervision of SS. BES, MLS, BMV, and DR supervised the work. MS analyzed the RAPD types using bioinformatics software. PS drafted the manuscript. SS, MS, SI, and NRH revised and finalized the manuscript for submission. All authors have read and approved the final manuscript.

## References

[1] Miller G.Y, Bartlett P.C, Lance S.E, Anderson J, Heider L.E (1993). Costs of clinical mastitis and mastitis prevention in dairy herds. J. Am. Vet. Med. Assoc.

[2] NAAS (2013). Mastitis Management in Dairy Animals, Policy Paper No. 61.

[3] Sharma N, Rho J.R, Hong Y.H, Kang T.Y, Lee H.K, Hur T.Y, Jeong D.K (2012). Bovine mastitis: An Asian perspective. Asian J. Anim. Vet. Adv.

[4] Thorberg B.M, Danielsson-Tham M.L, Emanuelson U, Waller K.P (2009). Bovine subclinical mastitis caused by different types of coagulase-negative staphylococci. J. Dairy Sci.

[5] Sampimon O.C, Lam T.J.G, Mevius D.J, Schukken Y.H, Zadoks R.N (2011). Antimicrobial susceptibility of coagulase-negative staphylococci isolated from bovine milk samples. Vet. Microbiol.

[6] Supré K, Haesebrouck F, Zadoks R.N, Vaneechoutte M, Piepers S, de Vliegher S (2011). Some coagulase-negative *Staphylococcus* species affect udder health more than others. J. Dairy Sci.

[7] Waller K.P, Aspan A, Nyman A, Persson Y, Andersson U.G (2011). CNS species and antimicrobial resistance in clinical and subclinical bovine mastitis. Vet. Microbiol.

[8] Gao J, Barkema H.W, Zhang L, Liu G, Deng Z, Cai L, Shan R, Zhang S, Zou J, Kastelic J.P, Han B (2017). Incidence of clinical mastitis and distribution of pathogens on large Chinese dairy farms. J. Dairy Sci.

[9] Zadoks R.N, Middleton J.R, Mcdougall S, Katholm J, Schukken Y.H (2011). Molecular epidemiology of mastitis pathogens of dairy cattle and comparative relevance to humans. J. Mammary Gland Biol. Neoplasia.

[10] Gillespie B.E, Headrick S.I, Boonyayatra S, Oliver S.P (2009). Prevalence and persistence of coagulase-negative *Staphylococcus* species in three dairy research herds. Vet. Microbiol.

[11] Piessens V, de Vliegher S, Verbist B, Braem G, van Nuffel A, de Vuyst L, Heyndrickx M, van Coillie E (2012). Intra-species diversity and epidemiology varies among coagulase-negative *Staphylococcus* species causing bovine intramammary infections. Vet. Microbiol.

[12] Naushad S, Barkema H.W, Luby C, Condas L.A, Nobrega D.B, Carson D.A, de Buck J (2016). Comprehensive phylogenetic analysis of bovine non-aureus staphylococci species based on whole-genome sequencing. Front. Microbiol.

[13] Reinoso E, Bettera S, Frigerio C, DiRenzo M, Calzolari A, Bogni C (2004). RAPD-PCR analysis of *Staphylococcus aureus* strains isolated from bovine and human hosts. Microbiol. Res.

[14] Bardiau M, Yamazaki K, Duprez J, Taminiau B, Mainil J.G, Ote I (2013). Genotypic and phenotypic characterization of methicillin-resistant *Staphylococcus aureus* (MRSA) isolated from milk of bovine mastitis. Lett. Appl. Microbiol.

[15] Chiang Y.C, Lai C.H, Lin C.W, Chang C.Y, Tsen H.Y (2014). Improvement of strain discrimination by the combination of superantigen profiles, PFGE, and RAPD for *Staphylococcus aureus* isolates from clinical samples and food-poisoning cases. Foodborne Pathog. Dis.

[16] Wang,D. Zhang, L, Zhou X, He Y, Yong C, Shen M, Szenci O, Han B (2016). Antimicrobial susceptibility, virulence genes, and randomly amplified polymorphic DNA analysis of *Staphylococcus aureus* recovered from bovine mastitis in Ningxia, China. J. Dairy Sci.

[17] Qu Y, Zhao H, Nobrega D.B, Cobo E.R, Han B, Zhao Z, Gao J (2018). Molecular epidemiology and distribution of antimicrobial resistance genes of *Staphylococcus* species isolated from Chinese dairy cows with clinical mastitis. J. Dairy Sci.

[18] Sundareshan S (2012). On Phenotypic and Molecular Characterization of Coagulase Negative Staphylococci Isolated from Clinical and Subclinical Cases of Bovine Mastitis, Ph.D. Thesis.

[19] Bauer A.W, Kirby M.M, Sherris J.C, Truck M (1966). Antibiotic susceptibility testing by a standardized single disk method. Am. J. Clin. Pathol.

[20] CLSI (2018). Performance Standards for Antimicrobial Disk and Dilution Susceptibility Tests for Bacteria Isolated from Animals, CLSI Supplement VET08.

[21] Hunter P.R, Gaston M.A (1988). Numerical index of the discriminatory ability of typing systems:An application of Simpson's index of diversity. J. Clin. Microbiol.

[22] Sawant A, Gillespie B, Oliver S (2009). Antimicrobial susceptibility of coagulase-negative *Staphylococcus* species isolated from bovine milk. Vet. Microbiol.

[23] Fessler A.T, Billerbeck C, Kadlec K, Schwarz S (2010). Identification and characterization of methicillin-resistant coagulase-negative staphylococci from bovine mastitis. J. Antimicrob. Chemother.

[24] Alnakip M.E, Quintela-Baluja M, Böhme K, Caamaño-Antelo S, Bayoumi M.A, Kamal R.M, Merwad A.M, Calo-Mata P, Velázquez J.B (2019). Molecular characterisation and typing the methicillin resistance of *Staphylococcus* spp. isolated from raw milk and cheeses in Northwest Spain:A mini-survey. Int. Dairy J.

[25] Fitzgerald J.R, Meaney W.J, Hartigan P.J, Smyth C.J, Kapur V (1997). Fine-structure molecular epidemiological analysis of *Staphylococcus aureus* recovered from cows. Epidemiol. Infect.

[26] Shimizu A, Kloos W.E, Berkhoff H.A, George C.G, Ballard D.N (1997). Pulsed-field gel electrophoresis of *Staphylococcus hyicus* and *Staphylococcus chromogenes* genomic DNA and its taxonomic, epidemiologic and ecologic applications in veterinary medicine. J. Vet. Med. Sci.

